# Reasons for hospitalization in HIV-infected children in West Africa

**DOI:** 10.7448/IAS.17.1.18818

**Published:** 2014-04-22

**Authors:** Fatoumata Dicko, Sophie Desmonde, Sikiratou Koumakpai, Hélène Dior-Mbodj, Fla Kouéta, Novisi Baeta, Niaboula Koné, Jocelyn Akakpo, Haby Signate Sy, Diarra Ye, Lorna Renner, Charlotte Lewden, Valériane Leroy, for the Pediatric IeDEA West Africa Working Group

**Affiliations:** 1 Service Pédiatrie Centre Hospitalier, Universitaire Gabriel Toure, Bamako, Mali; 2 Inserm, Centre Inserm U897 – Epidémiologie – Biostatistiques, University of Bordeaux, Bordeaux, France; 3 University of Bordeaux, ISPED, Centre Inserm U897 – Epidémiologie – Biostatistiques, Bordeaux, France; 4 Service Pédiatrie, Centre National Hospitalier Universitaire, Cotonou, Bénin; 5 Hopital pour Enfants Albert Royer, Dakar, Sénégal; 6 Service Pédiatrie, Hopital Général de Gaulle, Ouagadougou, Burkina Faso; 7 Korle Bu Teaching Hospital, Accra, Ghana

**Keywords:** HIV, paediatrics, hospitalization, Africa, morbidity, infectious diseases

## Abstract

**Introduction:**

Current knowledge on morbidity and mortality in HIV-infected children comes from data collected in specific research programmes, which may offer a different standard of care compared to routine care. We described hospitalization data within a large observational cohort of HIV-infected children in West Africa (IeDEA West Africa collaboration).

**Methods:**

We performed a six-month prospective multicentre survey from April to October 2010 in five HIV-specialized paediatric hospital wards in Ouagadougou, Accra, Cotonou, Dakar and Bamako. Baseline and follow-up data during hospitalization were recorded using a standardized clinical form, and extracted from hospitalization files and local databases. Event validation committees reviewed diagnoses within each centre. HIV-related events were defined according to the WHO definitions.

**Results:**

From April to October 2010, 155 HIV-infected children were hospitalized; median age was 3 years [1–8]. Among them, 90 (58%) were confirmed for HIV infection during their stay; 138 (89%) were already receiving cotrimoxazole prophylaxis and 64 children (40%) had initiated antiretroviral therapy (ART). The median length of stay was 13 days (IQR: 7–23); 25 children (16%) died during hospitalization and four (3%) were transferred out. The leading causes of hospitalization were WHO stage 3 opportunistic infections (37%), non-AIDS-defining events (28%), cachexia and other WHO stage 4 events (25%).

**Conclusions:**

Overall, most causes of hospitalizations were HIV related but one hospitalization in three was caused by a non-AIDS-defining event, mostly in children on ART. HIV-related fatality is also high despite the scaling-up of access to ART in resource-limited settings.

## Introduction

In 2010, the HIV/AIDS epidemic led to 1.8 million deaths worldwide, 14% of which occurred in children below 15 years of age [1]. Although substantial progress has been made in the management of paediatric HIV infection with the implementation of mother-to-child-prevention programmes and subsequently the introduction of antiretroviral therapy (ART), an estimated 3.3 million children are still living with HIV, of whom 3.1 million live in sub-Saharan Africa [1,2]. Before highly active ART was introduced, HIV-infected infant mortality was high and premature, reaching 35% in the first 12 months of life and 52% within 2 years [3]. Since 2004, following the introduction of ART, reductions in mortality and morbidity among HIV-infected children have been reported [4–8]. Nevertheless, the management of HIV-infected children remains challenging in resource-limited settings, particularly in West Africa [9]. First, the accurate identification of HIV-exposed children is largely insufficient [10]. Second, early infant diagnosis requires expensive and sophisticated molecular diagnosis techniques [11]; consequently, children are diagnosed at a late age, with an advanced clinical and/or immunological stage of HIV disease. Moreover, several studies have reported that advanced immunosuppression at ART initiation increases the risk of immune reconstitution syndrome [12,13]. Third, paediatric formulations are limited and the use of poorly tolerated regimens may expose to adverse events and treatment interruptions [14]. Fourth, severe morbidity in HIV-infected children is frequent whether they are ART-treated or not [15,16]. Finally, in case of occurrence of morbidity, resources to healthcare are often limited for financial reasons: besides ART, which is freely delivered to children, the costs of diagnosis tools and treatment for co-morbid events are borne by the children's families [17].

Current knowledge on morbidity and mortality comes mainly from data collected in specific research programmes, which may offer a different standard of care than that offered outside of a research context, where means for diagnosis and treatment are more limited. Hospitalization represents an important outcome measure, as a reliable indicator for morbidity and mortality, as well as healthcare utilisation.

In this study, we described hospitalization in tertiary hospital wards from a large prospective observational cohort of HIV-infected and HIV-exposed children in the paediatric West African International epidemiological Database to Evaluate AIDS (IeDEA) Collaboration.

## Methods

### Setting

The IeDEA network (http://www.iedea.org/) was initiated in July 2006 to address evolving questions in HIV/AIDS research, unanswerable by single cohorts, by collecting and harmonizing data from multiple HIV/AIDS cohorts and programmes. In 2012, the paediatric IeDEA West Africa collaboration (pWADA) involved 11 paediatric HIV/AIDS clinics spread out over seven countries in West Africa.

Health facilities in the IeDEA network deliver a model of paediatric care covering HIV diagnosis and treatment, including ART and prophylaxis of opportunistic infections. According to the national guidelines in the five included countries, HIV-exposed infants (known to be born to an HIV-infected mother) were supposed to routinely receive cotrimoxazole prophylaxis from six weeks of age, then an EID based on virological methods, which needs to be confirmed by serology from 15 months after the end of breastfeeding in breastfed children. HIV-infected children are typically seen at least every three months, and CD4 counts are measured every six months; ART, cotrimoxazole prophylaxis and blood analyses are free of charge. Children initiated ART according to the 2008 WHO recommendations, contemporaneous with the practices at time of ART initiation [18]: all HIV-infected infants diagnosed<12 months, irrespective of CD4 count or clinical stage, or according to clinical and immunological criteria in older children. Furthermore, children aged<18 months who meet presumptive diagnosis criteria were eligible for ART initiation.

### Design

We performed a six-month cross-sectional multicentre survey from April to October 2010 in five HIV-specialized paediatric hospital wards involved in the pWADA IeDEA collaboration: the Gabriel Touré Teaching Hospital (Bamako, Mali), the National Teaching Hospital (Cotonou, Benin), the Albert Royer Children's Hospital (Dakar, Senegal), the Général de Gaulle Teaching Hospital (Ouagadougou, Burkina Faso) and the Korle Bu Teaching Hospital (Accra, Ghana). All newly hospitalized HIV-infected children≤18 years, previously known as HIV-infected patients (HIV-1, HIV-2 or HIV-1+2) or diagnosed as HIV-infected during hospitalization were eligible, regardless of ART.

### Data collection

Baseline and follow-up information during hospitalization were recorded using a standardized clinical form. Data were extracted from hospitalization medical records and local databases. The following variables were recorded: social-demographic data (gender, data of birth, town, parents’ vital status, schooling); data of HIV diagnosis, type and circumstances of diagnosis, tuberculosis history; ART history (date of ART initiation, regimen, weight, height, WHO stage, CD4 and viral load - when available – current ART regimen); current prophylaxis; co-morbidity history, immunization status; date of hospitalization, cause, date of first symptoms, weight, height, WHO stage, CD4 count and viral load – when available – complete blood counts; diagnosis and type of diagnosis (clinical or presumptive), degree of severity; date of end of hospitalization, vital status at exit, underlying cause of death in that event. In case of hospitalization, the available diagnosis tools were provided to children's family. Unfortunately there were no data on prior PMTCT services on care as HIV-exposed infants. However, a proxy for identified HIV-exposed infants could be assumed from the children who were already on cotrimoxazole among those newly diagnosed.

### Clinical diagnosis

Ad-hoc event validation committees reviewed diagnoses within each centre. HIV-related events were defined according to the WHO definitions [19]. In this study, we defined malnutrition using Z-scores. Malnutrition is defined by a Z-score<−2 SD, with moderate malnutrition between −3 and −2 SD and severe malnutrition<−3 SD. Three indicators are defined: Weight-for-Age (WAZ) for underweight, Height-for-Age (HAZ) for stunting, and Weight-for-Height/BMI-for-Age (WHZ/BAZ) for wasting. “Cachexia” refers to unexplained severe underweight, stunting or wasting not adequately responding to standard therapy. “Underweight” refers to unexplained moderate malnutrition also defined by the WHO case definitions [19].

Immunodeficiency was defined according to age at time of CD4 measurement for those with available data, according to WHO definitions [19]. Immune reconstitution inflammatory syndrome (IRIS) was defined by the following criteria: HIV-infected, receiving ART with decreasing HIV-1 RNA levels and increasing CD4 cell counts since baseline, presenting clinical symptoms consistent with IRIS and a clinical evolution inconsistent with expected outcomes of previously diagnosed morbidity or drug toxicity within several weeks of starting ART [20]. Causality assessment of side effects [21] was defined with simplified categories adapted from the WHO-UMC causality categories [22]. Three categories were defined to describe the causality: (i) yes (reasonable time relationship to drug intake); (ii) no (time to drug intake inconsistent or other drugs can provide plausible explanations) and (iii) unknown (insufficient or contradictory information).

In order to determine one underlying cause for each hospitalization, the following priorities were applied if a patient had multiple diagnoses: (1) WHO stage 4 opportunistic diseases, (2) WHO stage 3 opportunistic diseases, (3) other non-AIDS-defining infections, (4) other cancerous events, (5) WHO stage 4 cachexia, (6) other diseases and (7) non-specific events including non-specific WHO stage 3 events (moderate malnutrition, persistent diarrhoea, persistent fever).

When a medical etiological diagnosis for hospitalization could not be identified, we reviewed diagnoses that comprised symptoms or clinical signs and attributed the hospitalization to those. Finally, we grouped the prioritized diagnoses associated with hospitalization into the following four categories: WHO stage 4 events (including cachexia); WHO stage 3 opportunistic infections; other non-AIDS-related infections; and other diseases or non-specific diseases (including non-specific WHO stage 3 events: moderate malnutrition, persistent diarrhoea, persistent fever). To determine the underlying cause of death, we used the International Classification of Diseases, Tenth Revision rules [23] and defined it as the disease or injury that initiated the morbid event(s) leading to death.

### Statistical analysis

We described the patterns and causes of hospitalization in children according to the WHO staging, knowledge of HIV infection at time of hospitalization, cotrimoxazole prophylaxis and ongoing ART at the time of hospitalization. Categorical data are presented as frequencies (percentage) and continuous variables as median [interquartile range (IQR)]. Proportions were compared by Fisher's exact tests.

## Results

### Population characteristics

In 2010, 1486 children were known to be HIV-infected and were followed up in these five clinics. From April to October 2010, 155 HIV-infected children were hospitalized once in one of the five participating tertiary hospitals in West Africa. Their main characteristics at entry to the hospital are presented in Table 1, according to age at time of hospitalization. Their median age at hospitalization was 3 years [IQR 1–8 years] and the sex ratio was 1. Overall, 99 children (64%) had reached WHO stage 4 of the disease and 138 (89%) were already receiving cotrimoxazole. The median time of hospitalization was 15 days (IQR: 11–23), during which 21% initiated ART. A large proportion of children were newly diagnosed during hospitalization (*n=*90, 58%); these children were aged in median 2 years (IQR: 1–6 years), 92% had reached WHO stage 3 or 4 of the disease, and 88% were already receiving cotrimoxazole prophylaxis (*n=*80), a proxy of HIV-exposure.

**Table 1:** Baseline characteristics of children hospitalized between April and October 2010 in West Africa, overall and according to the underlying cause. IeDEA Pediatric West Africa

	Overall *N=*155	<2 years *N=*63	2–5 years *N=*25	5–10 years *N=*42	10–15 years *N=*25
Median age (years), IQR	3 (1–8)	1 (1–1)	3 (2–3)	7 (6–8)	11 (10–13)
Female, n (%)	77 (50)	32 (51)	12 (48)	24 (57)	9 (36)
Time of HIV diagnosis, n (%)					
Prior to hospitalization	62 (40)	15 (24)	11 (44)	21 (50)	15 (60)
During hospitalization	90 (58)	46 (73)	14 (56)	20 (48)	10 (40)
Unknown	3 (2)	2 (3)	0 (0)	1 (2)	0 (0)
Median time since HIV diagnosis, months (IQR)					
Prior to hospitalization	6.8 (2.0–31.2)	2.2 (1.2–4.0)	6.3 (0.4–30.8)	17.7 (3.1–31.2)	41.5 (2.1–75.2)
Overall	0 (0–2.9)	0 (0–0)	0 (0–2.7)	0.2 (0.0–17.7)	2.0 (0.0–43.1)
Cotrimoxazole prophylaxis, n (%)	138 (89.0)	58 (92)	22 (88)	35 (83)	23 (92)
Antiretroviral treatment, n (%)					
Yes	64 (41)	21 (33)	8 (32)	23 (55)	12 (48)
No	90 (58)	42 (67)	17 (68)	18 (43)	13 (52)
Unknown	1 (1)	0 (0)	0 (0)	1 (2)	0 (0)
Timing of ART initiation, n (%)*					
Prior to hospitalization	36 (56)	9 (43)	4 (50)	13 (57)	10 (83)
During hospitalization	26 (41)	11 (52)	3 (38)	10 (43)	2 (17)
Unknown	2 (3)	1 (5)	1 (12)	0 (0)	0 (0)
Median time on ART months (IQR)*					
Prior to hospitalization	9.8 (1.6–32.7)	2.1 (1.5–3.3)	8.7 (3.9–17.0)	10.8 (3.7–31.3)	37.5 (6.2 –65.9)
Overall	0.9 (0–10.8)	0 (0–1.8)	0.3 (0–9.8)	1 (0–24.0)	32.7 (0.8–54.7)
ART eligibility among those not on ART, n (%)					
Eligible	88 (98)	42 (100)	17 (100)	17 (94)	12 (92)
Not eligible	0 (0)	0 (0)	0 (0)	0 (0)	0 (0)
Unknown	2 (2)	0 (0)	0 (0)	1 (6)	1 (8)
WHO stage at entry, n (%)					
4	99 (64)	48 (76)	16 (64)	23 (55)	12 (48)
3	42 (27)	12 (19)	5 (20)	16 (38)	9 (36)
2 or 1	14 (9)	3 (5)	4 (16)	3 (7)	4 (16)
Latest CD4%					
CD4 available, n (%)	38 (25)	14 (22)	5 (20)	11 (26)	8 (32)
Median CD4% (IQR)	14 (7–23)	16 (6–29)	18 (16–20)	14 (5–16)	11 (6–18)
Median time since latest measurement, months (IQR)					
Latest absolute CD4 count					
CD4 available, n (%)	71 (46)	20 (32)	14 (56)	25 (60)	12 (48)
Median CD4 count, cells/µL (IQR)	322 (112–847)	426 (232–718)	793 (382–1006)	283 (57–492)	171 (60–284)
Underlying cause of hospitalization, n (%)					
WHO stage 4 event	38 (25)	12 (19)	9 (36)	11 (26)	6 (24)
WHO stage 3 event	58 (37)	26 (41)	8 (32)	17 (40)	7 (28)
Other infections	43 (28)	18 (29)	4 (16)	11 (26)	9 (36)
Other diseases	16 (10)	7 (11)	4 (16)	3 (7)	3 (12)
Length of hospitalization (days), median (IQR)	13 (7–23)	12 (9–20)	16 (3–25)	15 (9–26)	11 (4–19)
Exit status					
Home	125 (81)	47 (75)	22 (88)	34 (81)	22 (88)
Deceased	25 (16)	15 (24)	2 (8)	6 (14)	2 (8)
Transferred to another hospital	4 (3)	1 (2)	1 (4)	1 (2)	1 (4)
Unknown	1 (0,6)	0 (0)	1 (0)	1 (3)	0 (0)

* Among those on ART.

Overall, 64 children were receiving ART of which 36 (56%) had already initiated treatment prior to hospitalization, 64% of which were aged>5 years; the median time on ART among those children was 9.8 months (IQR: 1.6–32). Among those who were not on ART at time of hospitalization, all (except two for whom eligibility could not be ascertained) were eligible according to 2008 WHO guidelines.

However, only 30% initiated treatment during hospitalization. These children were similar to those who did not initiate ART in terms of age, stage of the disease and cause of hospitalization. However, the median length of stay was significantly higher in those initiating ART (24.5 days, IQR: 15–42) compared to those who did not (13 days, IQR: 9–20, *p*<0.001).

Overall, 25 children (16%) died during hospitalization. These children were hospitalized for a significantly shorter period of time (9 days, IQR: 3–16) compared to those who remained alive (14 days, IQR: 9–23; *p=*0.007). Baseline characteristics were similar between both groups as was the distribution of events (see Table 1).

### Hospitalizations and diagnoses

The median length of stay was 13 days (IQR: 7–23 days); 25 children (16%) died during hospitalization, and four (3%) were transferred out to another ward or to another hospital. The most frequent causes of hospitalizations were: WHO stage 3 opportunistic infections (37%), other non-AIDS-defining infections (28%), WHO stage 4 events including cachexia (25%) and other diseases including WHO stage 3 non-specific diseases (10%). Among the non-AIDS-defining infections, the most frequent diagnoses were the following: infectious diarrhoea (31%), malaria (24%) and pneumonia (15%). Two hospitalizations were related to suspected adverse events to ART: one child presented severe anaemia and another, a suspected muco-cutaneous disease.

Many children were hospitalized with a second or third diagnosis associated with the main cause for hospitalization, yielding a total of 305 diagnoses, as described in Table 2. Overall, 53.1% of the diagnoses were attributable to infectious diseases, 43.9% to non-specific diseases and 3% to other diseases. Among infectious diseases, 34% were non-AIDS-defining events. When considering all 305 diagnoses, the five most frequent were the following: pneumonia (18%), cachexia (12.5%), underweight or malnutrition (11.8%), anaemia (9.8%), tuberculosis (6.6%); furthermore, 4.3% was malaria. Overall, 27.5% of the morbidity concerned non-AIDS-defining events.

**Table 2:** Distribution of the 305 diagnoses among HIV-infected children hospitalized in West Africa between April and October 2010. IeDEA Pediatric West Africa

			AIDS-defining events								
							
			Overall		Stages 1 and 2	Stage 3	Stage 4	Suspected adverse events		Definite diagnoses	
			
	N	%	N	%^a^	N	N	N	N	%	N	%^a^
Overall	305	100.0	221	72.5	10	141	70	3	1.0	249	81.6
Infectious diseases	162	53.1	107	66.0	6	74	27	0	0.0	113	69.8
Fungal diseases	14	4.6	14	100.0	0	8	6	0	0.0	10	71.4
Cryptococcosis	3	1.0	3	100.0	0	0	3	0	0.0	2	66.7
Oesophageal candidiasis	3	1.0	3	100.0	0	0	3	0	0.0	1	33.3
Oral candidiasis	8	2.6	8	100.0	0	8	0	0	0.0	7	87.5
Parasitic diseases	18	5.9	5	27.8	0	0	5	0	0.0	16	88.9
Malaria	13	4.3	0	0.0	0	0	0	0	0.0	12	92.3
Pneumocystosis (PCP)	2	0.7	2	100.0	0	0	2	0	0.0	2	100.0
Toxoplasmosis	3	1.0	3	100.0	0	0	3	0	0.0	2	66.7
Bacterial or other invasive infectious diseases	130	42.6	88	67.7	6	66	16	0	0.0	87	66.9
Infectious diarrhoea	15	4.9	0	0.0	0	0	0	0	0.0	5	33.3
Tuberculosis	20	6.6	20	100.0	0	15	5	0	0.0	2	10.0
Non-typhoidal salmonella	1	0.3	0	0.0	0	0	0	0	0.0	0	0.0
Typhoid fever	1	0.3	0	0.0	0	0	0	0	0.0	1	100.0
Pneumonia	55	18.0	45	81.8	0	45	0	0	0.0	48	87.3
Acute respiratory disease	6	2.0	6	100.0	0	6	0	0	0.0	5	83.3
Pleurisy	2	0.7	0	0.0	0	0	0	0	0.0	2	100.0
Meningitis	7	2.3	5	71.4	0	0	5	0	0.0	6	85.7
Lower urinary infection	1	0.3	0	0.0	0	0	0	0	0.0	1	100.0
Balanitis	1	0.3	0	0.0	0	0	0	0	0.0	1	100.0
Osteomyelitis	1	0.3	1	100.0	0	0	1	0	0.0	1	100.0
Otitis	14	4.6	5	35.7	5	0	0	0	0.0	10	71.4
Angular chelitis	1	0.3	1	100.0	1	0	0	0	0.0	1	100.0
Recurrent bacterial infections	5	1.6	5	100.0	0	0	5	0	0.0	4	80.0
Non-specific diseases	134	43.9	108	80.6	3	67	38	3	1.4	130	97.0
Cachexia	38	12.5	38	100.0	0	0	38	0	0.0	37	97.4
Weight loss/Malnutrition or denutrition	36	11.8	29	80.6	0	29	0	0	0.0	36	100.0
Fever	1	0.3	1	100.0	0	1	0	0	0.0	1	100.0
Diarrhoea	13	4.3	9	69.2	0	9	0	0	0.0	13	100.0
Anaemia	30	9.8	28	93.3	0	28	0	2	0.7	30	100.0
Thrombopaenia	1	0.3	0	0.0	0	0	0	1	0.3	1	100.0
Electrolytic disorder	2	0.7	0	0.0	0	0	0	0	0.0	2	100.0
Dehydration	10	3.3	0	0.0	0	0	0	0	0.0	7	70.0
Lymphadenopathy	3	1.0	3	100.0	3	0	0	0	0.0	3	100.0
Other diseases	9	3.0	6	66.7	1	0	5	0	0.0	6	66.7
Cholecystitis	1	0.3	0	0.0	0	0	0	0	0.0	1	100.0
Hemiplegia	1	0.3	0	0.0	0	0	0	0	0.0	1	100.0
Nephropathy	1	0.3	1	100.0	0	0	1	0	0.0	1	100.0
HIV encephalopathy	4	1.3	4	100.0	0	0	4	0	0.0	1	25.0
Parotide enlargement	1	0.3	1	100.0	1	0	0	0	0.0	1	100.0
Muco-cutaneous disease	1	0.3	0	0.0	0	0	0	0	0.0	1	100.0

a Row percentages.

The majority of the diagnoses were definite (81.6%). Of the 50 presumptive diagnoses, 40% were suspected cases of tuberculosis (of the 20 reported tuberculosis events, only two were definite according the WHO classification), and 6% were suspected HIV encephalopathy (one case out of four was definite).

Table 3 presents the causes of hospitalization according to the knowledge of HIV infection at time of hospitalization. We observed no significant difference in the overall distribution of AIDS-defining and non-defining events in children who were either diagnosed prior to hospitalization or those newly diagnosed. We observed a significantly higher proportion of children hospitalized for cachexia and weight loss among those newly diagnosed (31.1 and 53.3% respectively) compared to those diagnosed prior to hospitalization (16.1 and 24.2%, respectively) (*p=*0.0383 and *p<*0.0010 for cachexia and weight loss, respectively).

**Table 3:** Causes of hospitalizations overall and for the six most frequent events, the knowledge of HIV infection at time of hospitalization in HIV-infected children in West Africa (*N =*155). IeDEA Pediatric West Africa.

	Known HIV infection prior to hospitalization		Newly diagnosed for HIV during hospitalization		
			
Event	N	%	N	%	*p*
Overall^a^	62	40.1	90	59.2	
WHO stage 4 events	15	24.2	22	24.5	0.3258
WHO stage 3 events	22	35.5	36	40.0	
Non-AIDS-defining infection	15	24.2	26	28.9	
Non-AIDS-defining disease	10	16.1	6	6.7	
Selected events					
Pneumonia	22	35.5	32	35.5	0.9999
Tuberculosis	5	8.1	15	16.7	0.1477
Cachexia	10	16.1	28	31.1	0.0383
Weight loss	15	24.2	48	53.3	<0.001
Malaria	5	8.1	8	8.9	0.9999
Infectious diarrhoea	5	8.1	10	11.1	0.5924

a Three children had an undetermined date of HIV diagnosis. Analyses were carried out in the remaining 152.

When comparing the overall causes by treatment groups at time of hospitalization (ART and cotrimoxazole, cotrimoxazole only or neither), we observed a significant difference in the distribution of events with a higher proportions of non-AIDS-defining events in those who were on both cotrimoxazole and ART compared to those receiving cotrimoxazole only or no treatment at all (*p=*0.0321) (Table 4). When studying more specifically the seven most frequent events, we did indeed observe an overall higher proportion of malaria in those on ART (12.5%) compared to those who were not (6.6% in those on cotrimoxazole only and 7.7% in those receiving no treatment at all). Inversely, we observed a trend towards higher proportions of AIDS-defining events such as pneumonia or tuberculosis in those receiving no ART. Furthermore, we reported higher proportions of children hospitalized for cachexia and weight loss in children not on ART compared to those who had initiated ART (*p<*0.001, respectively). We also note that hospitalizations for cachexia were highest among children receiving no treatment at all (61.5%) compared to those on cotrimoxazole only (26.4%) and those on both ART and cotrimoxazole (6.3%) (*p<*0.001).

**Table 4:** Causes of hospitalizations overall and for the six most frequent events, according to treatment in HIV-infected children in West Africa (*N=*155). IeDEA Pediatric West Africa

	ART and cotrimoxazole prior to hospitalization		Cotrimoxazole only prior to hospitalization		No treatment prior to hospitalization		
				
Event	*N*	%	*N*	%	*N*	%	*p*
Overall	32	100.0	106	100.0	13	100.0	
WHO stage 4 events	9	28.1	22	20.8	6	46.2	0.0321
WHO stage 3 events	6	18.8	47	44.3	3	23.1	
Non-AIDS-defining infection	10	31.3	27	25.5	4	30.8	
Non-AIDS-defining disease	7	21.9	10	9.4	0	0.0	
The seven most frequent diagnoses among the above							
Pneumonia	7	21.9	41	38.7	5	38.5	0.2057
Tuberculosis	1	3.1	16	15.1	2	15.4	0.1757
Cachexia	2	6.3	28	26.4	8	61.5	<0.001
Weight loss	3	8.3	54	50.9	7	53.8	<0.001
Malaria	4	12.5	7	6.6	1	7.7	0.0608
Infectious diarrhoea	3	9.4	11	10.4	1	7.7	0.9999

Among the 25 children who died during hospitalization, the most frequent underlying causes of death were: cachexia (*n=*6), acute respiratory disease (*n=*6) and chronic diarrhoea (*n=*3) (Table 5).

**Table 5:** Distribution of the underlying causes of hospitalization and death in the 155 HIV-infected children hospitalized in West Africa. IeDEA Pediatric West Africa

	Cause of hospitalization		Cause of death	
		
Event	N	%	N	%
WHO stage 2 events	1	0.65	0	0
WHO 3 non-specific events	10	6.45	0	0
WHO 3 severe events	58	37.42	11	44
WHO stage 4 events	29	18.71	10	40
WHO stage 4 cachexia	9	5.81	0	0
Non-AIDS-defining infection	42	27.10	4	16
Non-AIDS-defining disease	3	1.94	0	0
Secondary effects to treatment	2	1.29	0	0
Uncategorised	1	0.65	0	0
Total	155	100.00	25	100

## Discussion

This cross-sectional study documents the causes of hospitalization in HIV-infected children in West Africa. We make several observations. First, of the 155 hospitalized children, 58% were unaware of their HIV status and were newly diagnosed during hospitalization; of these children, all were eligible for ART initiation and less than a third initiated treatment. Second, 62% of causes were AIDS-related, highlighting the advanced stage of HIV disease in these children at the time of hospitalization. Furthermore, we showed that among all of the events, 53% were in relation to an infection, underlining a context of residual infectious morbidity, despite cotrimoxazole prophylaxis and ART initiation. Indeed, the proportion of AIDS-defining causes of hospitalization was lower in children who were on both ART and cotrimoxazole. However, these children evidently still experience a substantial amount of non-AIDS-defining events serious enough to cause hospitalization.

The median age at hospitalization was 3 years, and we showed that hospitalization was an opportunity to diagnose 58% of HIV cases in this study. The fact that most of these newly HIV diagnosed children were already receiving cotrimoxazole prophylaxis at the time of their hospitalization indicated that they were already identified as HIV-exposed infants, but they had not yet been tested for HIV or had not been informed of their status. This reflects the flaws in the prevention of mother-to-child transmission of HIV (PMTCT) services in resource-limited settings and many missed opportunities for early HIV postnatal diagnosis and care. Indeed, a large proportion of HIV-infected infants never enters the pathway to early HIV care and is diagnosed at a late age, once symptomatic, initiating ART too late. This leads to severe morbidity and mortality [7,15,24,25]. Although early infant diagnosis is challenging in resource-limited settings for many reasons, including costs [26], it provides substantial benefits to HIV-exposed children and their families [27]. More efforts should be made when implementing early infant diagnosis in West Africa [28]. In addition, we highlighted the missed opportunities for treatment initiation in HIV-infected children eligible for ART in both populations: those who were already known to be HIV-infected prior to hospitalization and those were newly diagnosed. This probably occurred since these children were most likely not assessed for immunological ART criteria, one of the obstacles in accessing ART. The 2013 guidelines have simplified this by recommending ART initiation in all children aged<5 years regardless of their immunological criteria.

Care interventions aiming at identifying the earliest possible HIV-infected infants and treating them according to current guidelines would drastically reduce costs of HIV care. Indeed, previous studies suggest that hospitalization constitutes 15–49% of total HIV care, particularly in children at the most advanced disease stage [29,30]. Unfortunately, we do not have cost data in the current IeDEA context to compare. However, a previous study in Côte d'Ivoire reported that severe morbidity (diagnoses, treatments and hospital stays) represented $8.8 per patient, equivalent to 50% of patient expenditures [17]. In a context where costs are often the barrier to better care [16], it is essential to develop effective care strategies and identify HIV-infected children as early as possible. Studies in Thailand and South Africa have shown that longer pre-ART care or early ART initiation incur lower inpatient care costs [31,32]. Early and universal free access to ART services would improve paediatric outcomes and costs impacts at programme level [9].

Many studies in resource-limited settings have described hospitalizations in HIV-infected children, but tend to be restricted to cause-specific hospitalizations or focused on HIV-exposed rather than HIV-infected children [33–38]. In this study, we provide an accurate description of all the severe morbidity (with 82% of definite diagnoses) leading to hospitalization in HIV-infected children. This survey was conducted in tertiary hospitals, which provided better specificity of diagnoses than those obtained in smaller health care facilities where diagnosis tools are not always available [16,39]. Nevertheless, the true burden of hospitalization of HIV-infected children may be under-estimated as many children may be admitted to district or regional hospitals or non-specialist paediatric wards. The distribution of causes of hospitalization did not differ between those on cotrimoxazole and those not. This may reflect the fact that cotrimoxazole in West Africa is not implemented consistently despite the clinical practice guidelines. On the other hand, the spectrum of diagnoses associated with hospitalization tended to change whether the patient was on or off ART at time of hospitalization. Indeed, those on ART seemed to present with less AIDS-defining events, but more non-specific events comparable to what has been reported in the general population [40–42]. Previous studies have reported decreases in the number of hospitalizations due to common AIDS-defining diseases in the ART era [31,43], compensated by the appearance of non-AIDS-defining diseases.

Overall, 72.5% of events were AIDS and 27.5% non-AIDS defining. Among AIDS-defining events, respiratory diseases, particularly **recurrent** pneumonia constituted a large proportion of the causes of hospitalizations, significantly larger in those who were not on ART. The burden of respiratory manifestations in HIV-infected children is well documented, and our results are consistent with those of previous studies, in both high-resource and limited-resource settings [34,44], including a study in Thailand where it was reported that 30% of hospitalizations were due to pneumonia [31]. In addition, a study conducted in Côte d'Ivoire showed that one-third of all morbidity events in HIV-infected children were respiratory manifestations, which then decreased dramatically with the introduction of ART [45]; in our study, we observed a trend towards lower proportions of pneumonia in children who had initiated ART. On the other hand, despite many studies reporting a protective effect of cotrimoxazole on the occurrence of pulmonary diseases [46], we observed no difference in those who had initiated cotrimoxazole prophylaxis compared to those not. This could be explained by an information bias: cotrimoxazole may have prescribed to the sickest and therefore the most vulnerable to pulmonary diseases compared to those not on cotrimoxazole.

Almost half of the hospitalizations were caused by cachexia or underweight. This is consistent with previously published data on HIV/AIDS and malnutrition. A cross-sectional study led in Kenya reported the prevalence of malnutrition in HIV-infected children to reach 40% in 2008 [47]. Another study, also led in Kenya, reported malnutrition to be the underlying cause of more than half of the inpatient morbidity in HIV-infected children [48]. In our study, we observed higher proportions of cachexia and malnutrition in children who had not yet initiated ART compared to others and in those receiving cotrimoxazole compared to those not. This is in line with a recent study within the CHAP randomized placebo-controlled trial, reporting lower decreases in WAZ and HAZ scores in children receiving prophylaxis compared to placebo group [49]. We also noted higher proportions of cachexia and weight loss among newly HIV diagnosed children. Other studies have reported this high prevalence of HIV among malnourished children [50]–malnutrition wards should be an entry point to HIV infant diagnosis complementary to the early infant diagnosis procedure recommended, but in children already advanced in their HIV disease progression.

Although ART-treated children were less affected by cachexia and underweight, 15% of ART-treated children were still hospitalized for those causes. Similar observations have been reported elsewhere, in the ARROW open label randomized trial led in Uganda and Zimbabwe, authors reported that one in nine children with advanced HIV required hospitalization for severe malnutrition after ART initiation [37]. Possible reasons for this include intestinal parasitic infestations, vitamin and micronutrient deficiencies [51]. In addition to increasing coverage of ART among HIV-infected children, interventions to improve poor nutrition status such as nutrition supplementation programmes, step-down convalescent care and nutritional rehabilitation for children post-hospital discharge are necessary [52].

The third leading cause of hospitalization in this study is malaria. Previous studies have reported protective effects of cotrimoxazole when studying HIV and malaria co-infection [53]; a recent systematic review reported that cotrimoxazole in HIV-infected individuals protects against malaria [54]. We do not observe this in hospitalized HIV-infected children. Also, other studies report a role in the ART regimen, and possible protective effect of lopinavir-based ART [55,56]. Unfortunately we do not have the sufficient data to compare the proportion of malaria-induced hospitalizations by ART regimen. However, we do observe a trend towards lower proportions of malaria among ART-treated children. Yet, cotrimoxazole prophylaxis and ART alone are insufficient and vector control such as bed nets remains an important component of prevention of malaria – wide-scale deployment of bed nets has contributed to the fall in malaria morbidity and mortality [57]; however, further efforts are necessary in order to increase their coverage and use.

Our study has several drawbacks. As we have discussed previously, this study was conducted in tertiary hospitals where the distribution of severe morbidity may differ from that in more rural settings. Furthermore, social barriers and stigma associated with HIV/AIDS often lead to delays in accessing care and receipt of HIV diagnosis. In the absence of ART, mortality is severe among infants<2 years [3], which combined with the late diagnoses issues, induces a selection bias in our study population. Many perinatally infected children may have died before diagnosis and treatment, underestimating the toll of hospitalizations we observed in this six-month period. Second, in the case where children were on ART for a short period of time, we fail to clearly identify which came first, ART or severe morbidity. We advise caution in the interpretation of our results in ART-treated children; some events may not be solemnly residual morbidity in a resource-limited setting. Finally, missing data arose as a result of the reality of healthcare services in the field in Africa, and the lack of follow-up in clinical examinations did not allow us to confirm all diagnoses, over-estimating in some cases the burden of certain diseases. Nevertheless, our study provides a thorough description of causes of hospitalization in HIV-infected children, according to specific gold standard definitions. Although dating from 2010, these data remain rare in West Africa. In addition, we feel that in a context where children continue to access HIV care at a late stage of the disease, these data reflect a valuable baseline survey on the morbidity in hospitalized HIV-infected infants. It would be relevant to reassess these indicators over time.

## Conclusion

Overall, over half the children were newly diagnosed with HIV underlining the urgent need to improve access to early HIV diagnosis and treatment. Furthermore, one hospitalization in two is related to infectious diseases and one hospitalization in three was caused by a non-AIDS-related event; this proportion was higher in children on ART, reflecting a context of prevalent severe morbidity that may be beyond the threshold of ART effectiveness. In addition to ART, efforts must continue to focus on increasing the availability of cotrimoxazole prophylaxis for all in order to reduce effectively clinical morbidity in HIV-exposed and HIV-infected children.

## Appendix

**Author information**

**The IeDEA West Africa Collaboration Study Group (as of April 29, 2013):**

**Participating sites** (*members of the **Steering Committee**, ^§^members of the **Executive Committee**):

***Benin, Cotonou:***

Adults: Djimon Marcel Zannou*, Carin Ahouada, Jocelyn Akakpo, Christelle Ahomadegbé, Jules Bashi, Alice Gougounon-Houéto, Angèle Azon-Kouanou, Fabien Houngbé, Jean Sehonou (CNHU Hubert Maga).

Paediatrics: Sikiratou Koumakpaï*^§^, Florence Alihonou, Marcelline d'Almeida, Irvine Hodonou, Ghislaine Hounhoui, Gracien Sagbo, Leïla Tossa-Bagnan, Herman Adjide (CNHU Hubert Maga).

***Burkina Faso:***

Adults: Joseph Drabo*, René Bognounou, Arnaud Dienderé, Eliezer Traore, Lassane Zoungrana, Béatrice Zerbo (CHU Yalgado, ***Ouagadougou***), Adrien Bruno Sawadogo*^§^, Jacques Zoungrana, Arsène Héma, Ibrahim Soré, Guillaume Bado, Achille Tapsoba (CHU Souro Sanou, *Bobo Dioulasso*).

Paediatrics: Diarra Yé*, Fla Kouéta, Sylvie Ouedraogo, Rasmata Ouédraogo, William Hiembo, Mady Gansonré (CH Charles de Gaulle, *Ouagadougou*).

***Côte d'Ivoire, Abidjan:***

Adults: Eugène Messou*, Joachim Charles Gnokoro, Mamadou Koné, Guillaume Martial Kouakou, (ACONDA-CePReF); Clarisse Amani Bosse*, Kouakou Brou, Achi Isidore Assi (ACONDA-MTCT-Plus); Henri Chenal*, Denise Hawerlander, Franck Soppi (CIRBA); Albert Minga*, Yao Abo, Jean-Michel Yoboue (CMSDS/CNTS); Serge Paul Eholié*^§^, Mensah Deborah Noelly Amego, Viviane Andavi, Zelica Diallo, Frédéric Ello, Aristophane Koffi Tanon (SMIT, CHU de Treichville), Serge Olivier Koule*, Koffi Charles Anzan, Calixte Guehi (USAC, CHU de Treichville);.

Paediatrics: Edmond Addi Aka*, Koffi Ladji Issouf, Jean-Claude Kouakou, Marie-Sylvie N'Gbeche, (ACONDA-CePReF); Touré Pety*, Divine Avit-Edi (ACONDA-MTCT-Plus); Kouadio Kouakou*, Magloire Moh, Valérie Andoblé Yao (CIRBA); Madeleine Amorissani Folquet*, Marie-Evelyne Dainguy, Cyrille Kouakou, Véronique Tanoh Méa-Assande, Gladys Oka-Berete, Nathalie Zobo, Patrick Acquah, Marie-Berthe Kokora (CHU Cocody); Tanoh François Eboua*, Marguerite Timité-Konan, Lucrèce Diecket Ahoussou, Julie Kebé Assouan, Mabéa Flora Sami, Clémence Kouadio (CHU Yopougon).

***Ghana, Accra:***

Paediatrics: Lorna Renner*^§^, Bamenla Goka, Jennifer Welbeck, Adziri Sackey, Seth Ntiri Owiafe (Korle Bu TH).

***Guinea-Bissau:***

Adults: Christian Wejse*^§^, Zacarias José Da Silva*, Joao Paulo (Bandim Health Project), The Bissau HIV cohort study group: Amabelia Rodrigues (Bandim Health Project), David da Silva (National HIV program Bissau), Candida Medina (Hospital National Simao Mendes, Bissau), Ines Oliviera-Souto (Bandim Health Project), Lars Østergaard (Dept of Infectious Diseases, Aarhus University Hospital), Alex Laursen (Dept of Infectious Diseases, Aarhus University Hospital), Morten Sodemann (Dept of Infectious Diseases, Odense University Hospital), Peter Aaby (Bandim Health Project), Anders Fomsgaard (Dept. of Virology, Statens Serum Institut, Copenhagen), Christian Erikstrup (Dept. of Clinical Immunology), Jesper Eugen-Olsen (Dept. of Infectious Diseases, Hvidovre Hospital, Copenhagen).

***Mali, Bamako:***

Adults: Moussa Y Maïga*^§^, Fatoumata Fofana Diakité, Abdoulaye Kalle, Drissa Katile (CH Gabriel Toure), Hamar Alassane Traore*, Daouda Minta*, Tidiani Cissé, Mamadou Dembelé, Mohammed Doumbia, Mahamadou Fomba, Assétou Soukho Kaya, Abdoulaye M Traoré, Hamady Traoré, Amadou Abathina Toure (CH Point G).

Paediatrics: Fatoumata Dicko*, Mariam Sylla, Alima Berthé, Hadizatou Coulibaly Traoré, Anta Koïta, Niaboula Koné, Clémentine N'Diaye, Safiatou Touré Coulibaly, Mamadou Traoré, Naïchata Traoré (CH Gabriel Toure).

***Nigeria:***

Adults: Man Charurat* (UMB/IHV), Samuel Ajayi*, Georgina Alim, Stephen Dapiap, Otu (UATH, *Abuja*), Festus Igbinoba (National Hospital *Abuja*), Okwara Benson*, Clément Adebamowo*, Jesse James, Obaseki, Philip Osakede (UBTH, *Benin City*), John Olasode (OATH, *Ile-Ife*).

***Senegal, Dakar:***

Adults: Moussa Seydi*, Papa Salif Sow, Bernard Diop, Noël Magloire Manga, Judicael Malick Tine^§^, Coumba Cissé Bassabi (SMIT, CHU Fann).

Paediatrics: Haby Signate Sy*, Abou Ba, Aida Diagne, Hélène Dior, Malick Faye, Ramatoulaye Diagne Gueye, Aminata Diack Mbaye (CH Albert Royer).

***Togo, Lomé:***

Adults: Akessiwe Patassi*, Awèrou Kotosso, Benjamin Goilibe Kariyare, Gafarou Gbadamassi, Agbo Komi, Kankoé Edem Mensah-Zukong, Pinuwe Pakpame (CHU Tokoin/Sylvanus Olympio).

Paediatrics: Koko Lawson-Evi*^§^, Yawo Atakouma, Elom Takassi, Améyo Djeha, Ayoko Ephoévi-gah, Sherifa El-Hadj Djibril (CHU Tokoin/Sylvanus Olympio).

**Executive Committee*:** François Dabis (Principal Investigator, Bordeaux, France), Emmanuel Bissagnene (Co-Principal Investigator, Abidjan, Côte d'Ivoire), Elise Arrivé (Bordeaux, France), Patrick Coffie (Abidjan, Côte d'Ivoire), Didier Ekouevi (Abidjan, Côte d'Ivoire), Antoine Jaquet (Bordeaux, France), Valériane Leroy (Bordeaux, France), Charlotte Lewden (Bordeaux, France), Annie J Sasco (Bordeaux, France).

**Operational and Statistical Team:** Dieudonné Amani (Abidjan, Côte d'Ivoire), Jean-Claude Azani (Abidjan, Côte d'Ivoire), Eric Balestre (Bordeaux, France), Serge Bessekon (Abidjan, Côte d'Ivoire), Franck Bohossou (Abidjan, Côte d'Ivoire), Camille Gilbert (Bordeaux, France), Sophie Karcher (Bordeaux, France), Jules Mahan Gonsan (Abidjan, Côte d'Ivoire), Jérôme Le Carrou (Bordeaux, France), Séverin Lenaud (Abidjan, Côte d'Ivoire), Célestin Nchot (Abidjan, Côte d'Ivoire), Karen Malateste (Bordeaux, France), Amon Roseamonde Yao (Abidjan, Côte d'Ivoire), Bertine Siloué (Abidjan, Côte d'Ivoire).

**Administrative Team:** Gwenaelle Clouet (Bordeaux, France), Madikona Dosso (Abidjan, Côte d'Ivoire), Alexandra Doring^§^ (Bordeaux, France), Adrienne Kouakou (Abidjan, Côte d'Ivoire), Elodie Rabourdin (Bordeaux, France), Jean Rivenc (Pessac, France).

**Consultants/Working Groups:** Xavier Anglaret (Bordeaux, France), Boubacar Ba (Bamako, Mali), Renaud Becquet (Bordeaux, France), Jean Bosco Essanin (Abidjan), Andrea Ciaranello (Boston, USA), Sébastien Datté (Abidjan, Côte d'Ivoire), Sophie Desmonde (Bordeaux, France), Jean-Serge Elvis Diby (Abidjan, Côte d'Ivoire), Geoffrey S.Gottlieb* (Seattle, USA), Apollinaire Gninlgninrin Horo (Abidjan, Côte d'Ivoire), Serge N'zoré Kangah (Abidjan, Côte d'Ivoire), Denis Malvy (Bordeaux, France), David Meless (Abidjan, Côte d'Ivoire), Aida Mounkaila-Harouna (Bordeaux, France), Camille Ndondoki (Bordeaux, France), Caroline Shiboski (San Francisco USA), Boris Tchounga (Abidjan, Côte d'Ivoire), Rodolphe Thiébaut (Bordeaux, France), Gilles Wandeler (Dakar, Senegal).

**Funding:** The National Cancer Institute (NCI), the Eunice Kennedy Shriver National Institute of Child Health & Human Development (NICHD) and the National Institute of Allergy and Infectious Diseases (NIAID) of the U.S. National Institutes of Health (NIH), as part of the International Epidemiologic Databases to Evaluate AIDS (IeDEA) under Award Number U01AI069919. The content is solely the responsibility of the authors and does not necessarily represent the official views of the National Institutes of Health.

**Coordinating Centre:** ISPED, Univ Bordeaux Segalen, Bordeaux, France

**Regional Office:** PAC-CI, Abidjan, Côte d'Ivoire

**Methodological Support**: MEREVA, Bordeaux, France

**Website:** http://www.mereva.net/iedea
